# The non-nutritive sweetener rebaudioside a enhances phage infectivity

**DOI:** 10.1038/s41598-025-85186-w

**Published:** 2025-01-08

**Authors:** Luigi Marongiu, Ewa Brzozowska, Jan Brykała, Markus Burkard, Herbert Schmidt, Bożena Szermer-Olearnik, Sascha Venturelli

**Affiliations:** 1 Department of Nutritional Biochemistry, University of Hohenheim, Garbenstraße 30, 70599 Stuttgart, Germany; 2 Department of Immunology of Infectious Diseases, Hirszfeld Institute of Immunology and Experimental Therapy, Polish Academy of Sciences, 12 R. Weigl St, Wroclaw, 53114 Poland; 3 Department of Food Microbiology, Institute of Food Science and Biotechnology, University of Hohenheim, Garbenstraße 28, 70599 Stuttgart, Germany; 4 Department of Experimental Oncology, Hirszfeld Institute of Immunology and Experimental Therapy, Polish Academy of Sciences, 12 R. Weigl St, Wroclaw, 53114 Poland; 5 Department of Vegetative and Clinical Physiology, Institute of Physiology, University of Tuebingen, Wilhelmstraße 56, 72074 Tuebingen, Germany

**Keywords:** Bacteriophages, Gastrointestinal diseases, Biofilms

## Abstract

Non-nutritive sweeteners (NNS) are widely employed in foodstuffs. However, it has become increasingly evident that their consumption is associated with bacterial dysbiosis, which, in turn, is linked to several health conditions, including a higher risk of type 2 diabetes and cancer. Among the NNS, stevia, whose main component is rebaudioside A (rebA), is gaining popularity in the organic food market segment. While the effect of NNS on bacteria has been established, the impact of these sweeteners on bacterial viruses (phages) has been neglected, even though phages are crucial elements in maintaining microbial eubiosis. The present study sought to provide a proof-of-concept of the impact of NNS on phage infectivity by assessing the binding of rebA to phage proteins involved in the infection process of enteropathogenic bacteria, namely the fiber protein gp17 of *Yersinia enterocolitica* phage φYeO3-12 and the tubular baseplate protein gp31 of *Klebsiella pneumoniae* phage 32. We employed docking analysis and a panel of in vitro confirmatory tests (microscale thermophoresis, *RedStarch*™ depolymerization, adsorption, and lysis rates). Docking analysis indicated that NNS can bind to both fiber and baseplate proteins. Confirmatory assays demonstrated that rebA can bind gp31 and that such binding increased the protein’s enzymatic activity. Moreover, the binding of rebA to gp17 resulted in a decrease in the adsorption rate of the recombinant protein to its host but increased the *Yersinia* bacteriolysis caused by the whole phage compared to unexposed controls. These results support the hypothesis that NNS can impair phage infectivity, albeit the resulting effect on the microbiome remains to be elucidated.

**Supplementary Information:**

The online version contains supplementary material available at 10.1038/s41598-025-85186-w.

## Introduction

The gut microbiota is a crucial modulator of human physiology^1^. A growing body of evidence supports the concept of a correlation between alterations in the relative abundances of microbes present in the gastrointestinal tract (GIT) compared to the composition observed in healthy conditions (a process known as dysbiosis) and a variety of pathologies such as diabetes, chronic inflammation, cancer, and even personality disorders^2^.

One of the primary regulators of GIT eubiosis comprises bacteriophages, or phages for short^3^. Phages can modulate the immune response, improve health, and contribute to maintaining GIT species richness^4,5^. Most phages are characterized by a tail, an injection system that is essentially a nanomachine involved in precise host recognition, receptor binding, and injection of the phage nucleic acids into the bacterial host cell^6–8^. Some tail proteins engage in the enzymatic breakdown of polysaccharides found in biofilm matrices or bacterial capsules, allowing the phage to access the inner membrane of Gram-negative bacteria and begin the infection process^9,10^.

Phage infectivity can also contribute to a decrease in the pathogenesis of invading gastrointestinal pathogens^11^. Among foodborne enteropathogenic bacteria are *Yersinia enterocolitica* and *Klebsiella pneumoniae*. In particular, *Y. enterocolitica* is increasingly reported to bear antibiotic-resistance traits and should be considered a relevant emerging zoonotic pathogen^12,13^. The species can be subdivided into several serotypes, with O:3 being one of the most prevalent in Europe^14^, causing asymptomatic infections in swine and being transmitted to humans by consuming undercooked meat^15^. Phages are now considered for their antibacterial properties against this bacterium to overcome the ineffectiveness of antibiotics in clearing yersinioses^16,17^. On the other hand, *K. pneumoniae* colonizes various organs, including the lungs, the urinary tract, blood, and liver, forming biofilms and expressing a capsule^18^.

Biofilms and capsules are mainly formed by extracellular polymeric substance (EPS) composed of exopolysaccharides, secreted proteins, lipids, and extracellular DNA^17^. EPS comprises repeating units of oligosaccharide with non-carbohydrate substitutes via glycosidic linkages, including those present in the disaccharide of maltose^19^. Phages are currently being explored as anti-biofilm and anti-microbial agents^17,20^.

Since hindrance to the phage infection process can alter the bacteriome^21,22^, determining how nutrients might affect GIT phage infectivity is critical for understanding the causes of dysbiosis and fine-tuning phage action. Such knowledge can guide a healthy diet, help removing pathobionts, reducing the virulence of invading bacterial pathogens, and improving antibacterial phage treatments^23,24^.

Non-nutritive sweeteners (NNS) are compounds widely used in foodstuffs and other commercial items such as mouthwashes and vitamin supplements^25^. The World Health Organization (WHO) recently revised its previous NNS safety claim and advised avoiding their consumption^23^. Despite such recommendations, NNS are still extensively present in our diet and their consumption has even increased in recent years^26–28^. Among the most popular NNS, there is stevia, which is formed by a combination of several steviol glycosides, chiefly rebaudioside A (rebA) but also steviol, stevioside, rebaudioside B (rebB), and steviolbioside (SB)^29^. Stevia is especially popular in the organic food business due to its natural origin and additional biological activities besides sweetening power^30,31^.

While a growing body of experimental evidence demonstrates that NNS can alter bacterial biochemistry, including the alteration of quorum sensing signals or enhancing the rate of horizontal gene transfer^32–34^, the effect of NNS on phage biology has been neglected. Nonetheless, it has been shown that several nutrients, such as fruit juices or sucrose, can modulate virion structure^35–37^. Specific phage proteins can bind to natural sugars. For instance, the tail tubular protein gp31 of phage *Klebsiella* phage (KP32) can bind the disaccharide maltose to produce the monosaccharide glucose through its inherent glycolytic activity, allowing the phage to penetrate biofilms and capsules^9^. Moreover, the phage T7’s tail protein gp17, which is closely related to *Y. enterocolitica* phage φYeO3-12’s gp17, recognizes maltose moieties present in the surface lipopolysaccharide (LPS) of its host^38–40^. Alterations in the binding of saccharides to these proteins can be expected to modify the phage adsorption rate by competing for the same binding pockets. The present study sought to provide exploratory in silico and in vitro experimental support for the hypothesis that NNS could interfere with phage infectivity. We focused our exploratory analysis on rebA and experimentally showed its impact on *Yersinia* and *Klebsiella* phages biological activity.

## Results

### In silico analysis of NNS binding to phage proteins

**Fig. 1:**
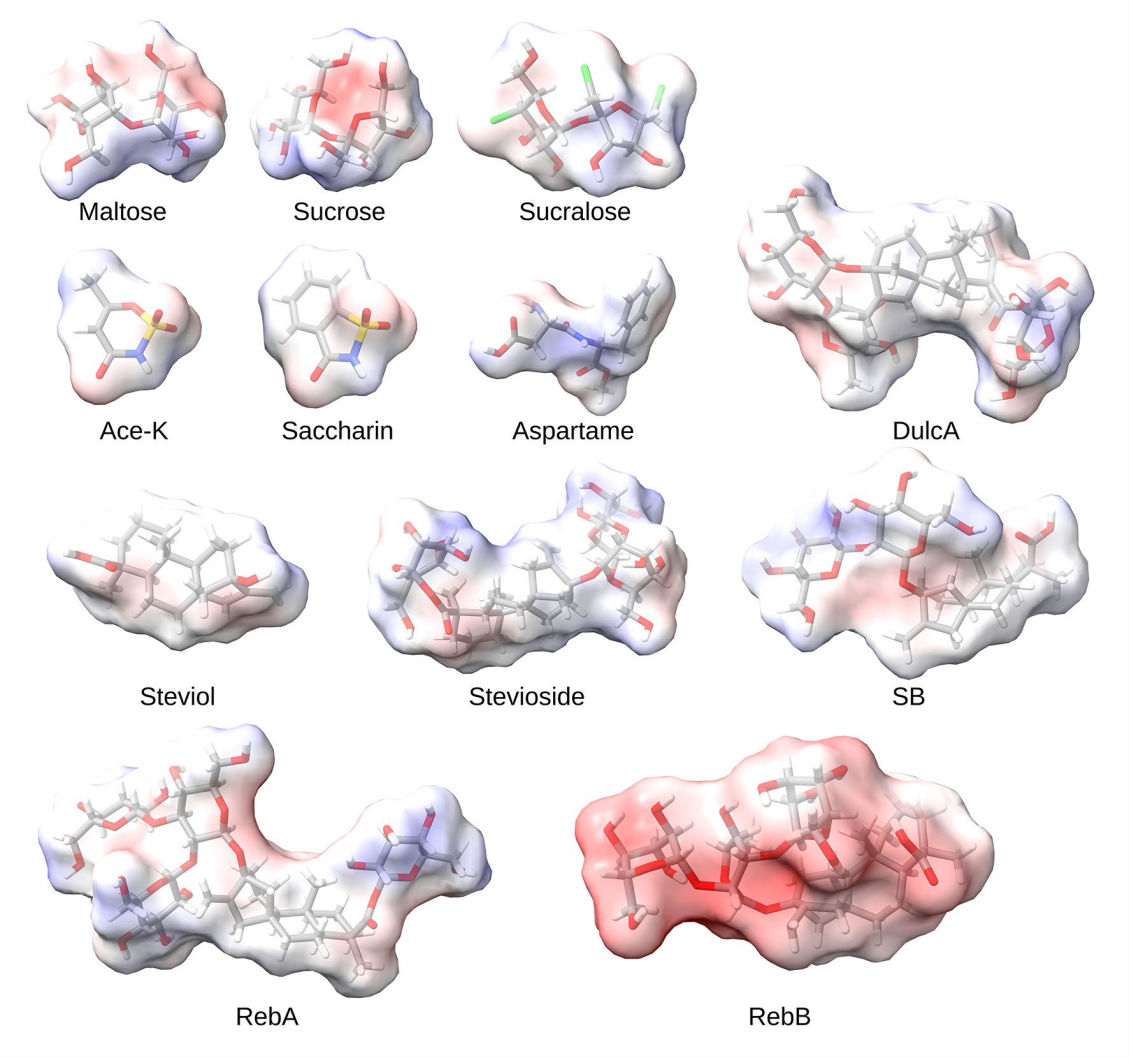
Structure of the NNS selected in the present study. Tridimensional structure of selected non-nutritive sweeteners (NNS) with superimposed molecular volume and color-coded by electrostatic potential (negative charges in red, positive charges in blue, neutral charges in white). Atoms are color-coded as follows: gray for carbon, white for hydrogen, red for oxygen, blue for nitrogen, yellow for sulfur, and green for chlorine. Acronyms: ace-K = acesulfame potassium; dulcA = dulcoside A; rebA/rebB = rebaudioside A/B; SB = steviolbioside.

Figure 1 reports the panel of NNS investigated for docking analysis, and Table 1 reports their physical properties. Table 2 lists the characteristics of the proteins used for docking, while Table 3 lists the residues engaged by the most stable poses overlapping with maltose and the corresponding binding energies. Taking advantage for the presence of a high-resolution structure for *K. pneumoniae* phage KP32 gp31 deposited in the Protein Data Bank, we performed docking analysis on this structure. Conversely, there is no data for *Y. enterocolitica* phage φYeO3-12 gp17; thus, to investigate the possible binding of NNS on phage tail proteins, we used phage T7 g17 as an alternative due to the high similarity of these proteins.

**Table 1:** Physical properties of the NNS selected in the present study.

Molecule	Area (nm^2^)	Volume (nm^3^)	MW^a^	pI
Acesulfame potassium (ace-K)	1.337	0.114	201.2	3.79
Aspartame	2.722	0.264	294.1	6.05
Dulcoside A (dulcA)	3.837	0.404	788.9	3.49
Maltose	2.591	0.281	342.3	4.00
Rebaudioside A (rebA)	7.061	0.894	967.0	3.92
Rebaudioside B (rebB)	4.756	0.514	804.9	3.92
Saccharin	1.507	0.135	183.2	7.83
Steviol	2.581	0.301	318.5	4.00
Steviolbioside (SB)	3.841	0.408	642.7	3.57
Stevioside	3.558	0.353	804.9	3.57
Sucralose	2.937	0.324	397.6	5.72
Sucrose	2.613	0.285	342.3	5.72

a) Molecular weight (Da).

**Table 2:** Characteristics of the viral proteins included in the present study.

Phage	Type	Protein	Name	PDB ID	Resolution^a^	Ref.
T7	Fiber	N-term.	gp17	4A0U	0.20 nm	^40^
KP32	Tail	Tail Tubular Protein A	gp31	5MU4	0.19 nm	^9^

a) From X-ray diffraction analysis.

**Table 3:** Most stable poses obtained by docking analysis.

Target	Ligand	Min. ΔG^a^	Ch. ΔG^b^	Bound residues^c^	Locus
gp17	Ace-K	−4.630	−4.169	(A) Gly_521_, Gly_522_	G521
	Aspartame	−5.917	−5.917	(C) Gly_521_, Gly_52B_	G521
	DulcA	−7.667	−7.667	(B) Arg_525_ (C) Gly_521_	G521
	Maltose	−5.148	−5.148	(A) Asn_501_, Gly_521_, Gly_522_	G521
	RebA	−7.038	−7.038	(A) Ser_519_, Gly_521_, Gly_522_ (C) Gly_521_	G521
	RebB	−8.703	−8.703	(A) Trp_523_, Arg_525_, Ser541 (C) Asn_501_, Asp_520_, Gly_521_, Ser_541_	G521
	Saccharin	−5.195	−5.083	(A) Gly_521_ (B) Trp_523_	G521
	Steviol	−6.831	−6.831	(A) Gly_522_ (B) Ser_519_	G521
	SB	−8.488	−8.488	(A) Arg_501_, Trp_523_ (B) Arg_525_, Gly_522_ (C) Trp_523_	G521
	Stevioside	−7.783	−7.783	(B) Gly_522_, Arg_525_	G521
	Sucralose	−5.386	−5.386	(B) Gly_522_ (C) Gly_521_	G521
	Sucrose	−5.503	−5.503	(B) Trp_523_ (C) Gly_522_	G521
gp31	Ace-K	−4.967	−4.967	(A) Arg_192_ (B) Arg_164_	K137
	Aspartame	−5.524	−5.524	(D) Glu_128_	S144
	DulcA	−8.339	−8.339	(B) Arg_52_, Ser_86_, Ile_87_, Tyr_92_	S144
	Maltose	−6.051	−6.051	(A) Gly_191_, Arg_192_ (B) Lys_137_, Arg_140_, Arg_164_	K137
	RebA	−8.249	−8.249	(A) Arg_49_, Arg_52_, Ser_56_, Lys_57_, Tyr_84_, Tyr_177_, Met_179_ (B) Lys_57_	K57
	RebB	−9.117	−9.117	(C) Arg_140_, Ser_144_, Glu_153_ (D) Asp_173_	S144
	Saccharin	−5.858	−5.858	(C) Ser_144_	S144
	Steviol	−7.500	−7.500	(A) Glu_150_	S144
	SB	−8.189	−8.189	(A) Met_179_ (B) Lys_57_	K57
	Stevioside	−8.869	−8.869	(A) Lys_137_, Arg_140_, Glu_153_ (C) Ser_144_	S144
	Sucralose	−6.070	−6.070	(D) Cys_129_	S144
	Sucrose	−6.065	−6.065	(A) Gly_148_ (D) Cys_129_	S144

a) Minimum ΔG among all poses.

b) Chosen ΔG of the pose binding the residues reported in the next column.

c) Letters in brackets refer to the chain involved in the binding.

### GP31

**Fig. 2:**
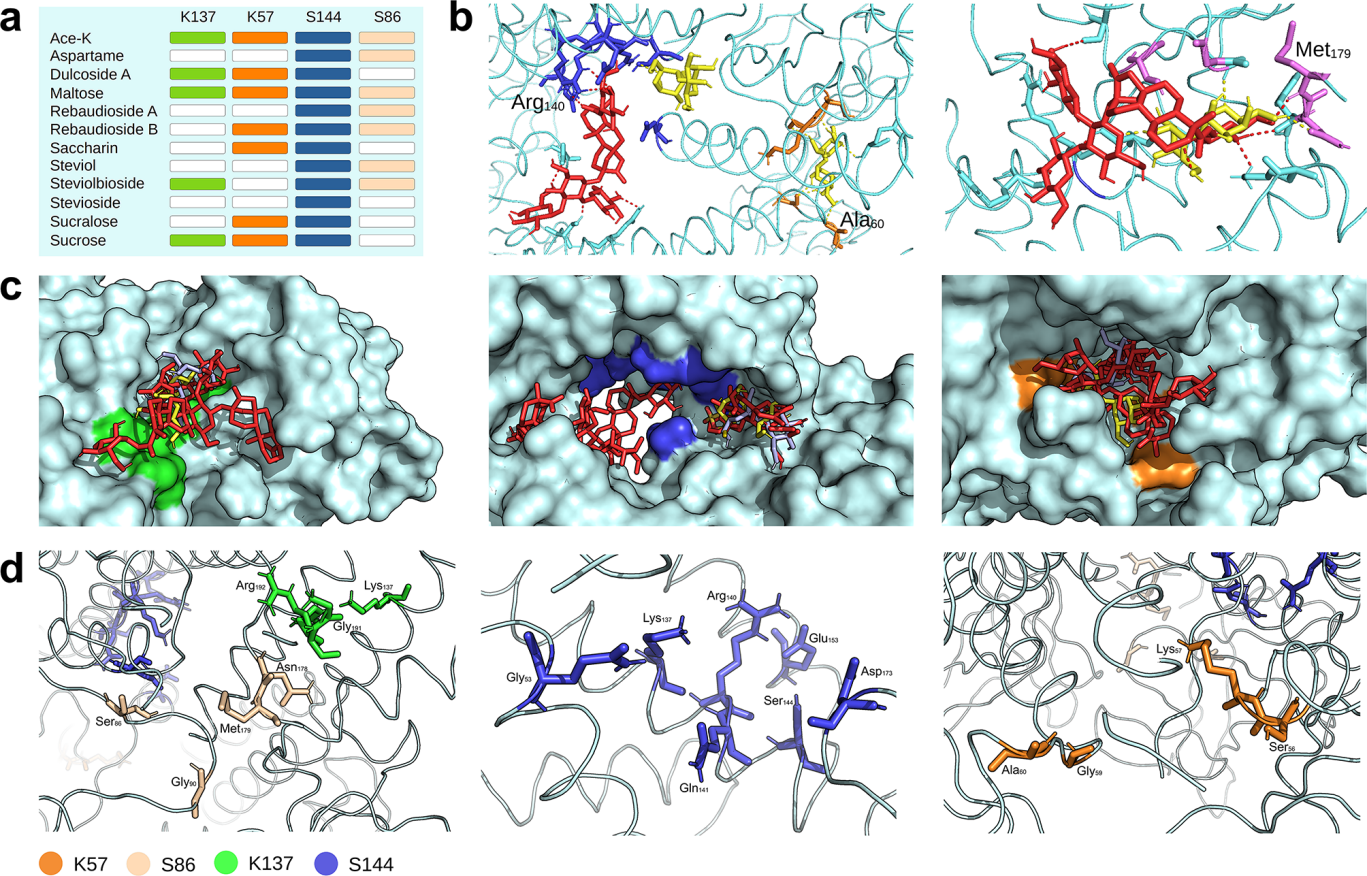
Docking analysis of gp31. (**a**) Summary table of the most stable poses between selected NNS and the baseplate gp31 of phage KP32. The panel reports the loci engaged by the NNS at different binding energies. To note that all NNS, as well as maltose, bound to the locus S144 that contained the catalytic residue Glu_153_. (**b**) Localization of rebA (red) and maltose (yellow) on gp31. On the left: Maltose can bind pockets that are not engaged by rebA (locus K57, residues highlighted in orange) or that do not directly overlap with rebA (locus S144, residues highlighted in blue). On the right: maltose and rebA can compete for the same pocket (locus S86, residues highlighted in magenta). Representative residues within these pockets are indicated. (**c**) Surface visualization of gp31 with the position of the NNS (in red) and the natural sugars maltose (in yellow) and sucrose (in lilac). To note that the loci are located in different positions within the protein. (**d**) Location of the residues engaged by the NNS. In panels (c) and (d), the residues are color-coded according to the legend reported at the bottom of the figure.

The NNS investigated herein bound to several residues of KP32 gp31, making it challenging to identify a special pocket for sweetener identification. Nonetheless, the selected NNS displayed poses that engaged pockets also used by maltose. However, those conformations were not necessarily the most stable (Fig. 2a). In particular, rebA could bind to pockets not directly engaged by maltose as well as residues directly overlapping maltose (Fig. 2b). Understanding the possible interaction between NNS and gp31 was further complicated because different protein chains could be engaged.

Despite the challenging outlook for the NNS/TTPA interaction, specific portions of the protein were more prone to creating bonds with NNS (Fig. 2c). Specifically, it was possible to identify four pockets that frequently formed bonds with NNS and maltose, named herein for convenience loci K57, S86, K137, and S144 because they were clustered around residues Lys_57_, Ser_86_ (both on chain D), Lys_137_ (chain B), and Ser_144_ (chain A), respectively; the latter locus included the catalytic residue Glu_153_^41^ (Fig. 2d). Notably, all NNS could bind to locus S144, although engaging different residues within the pocket.

### GP17

**Fig. 3:**
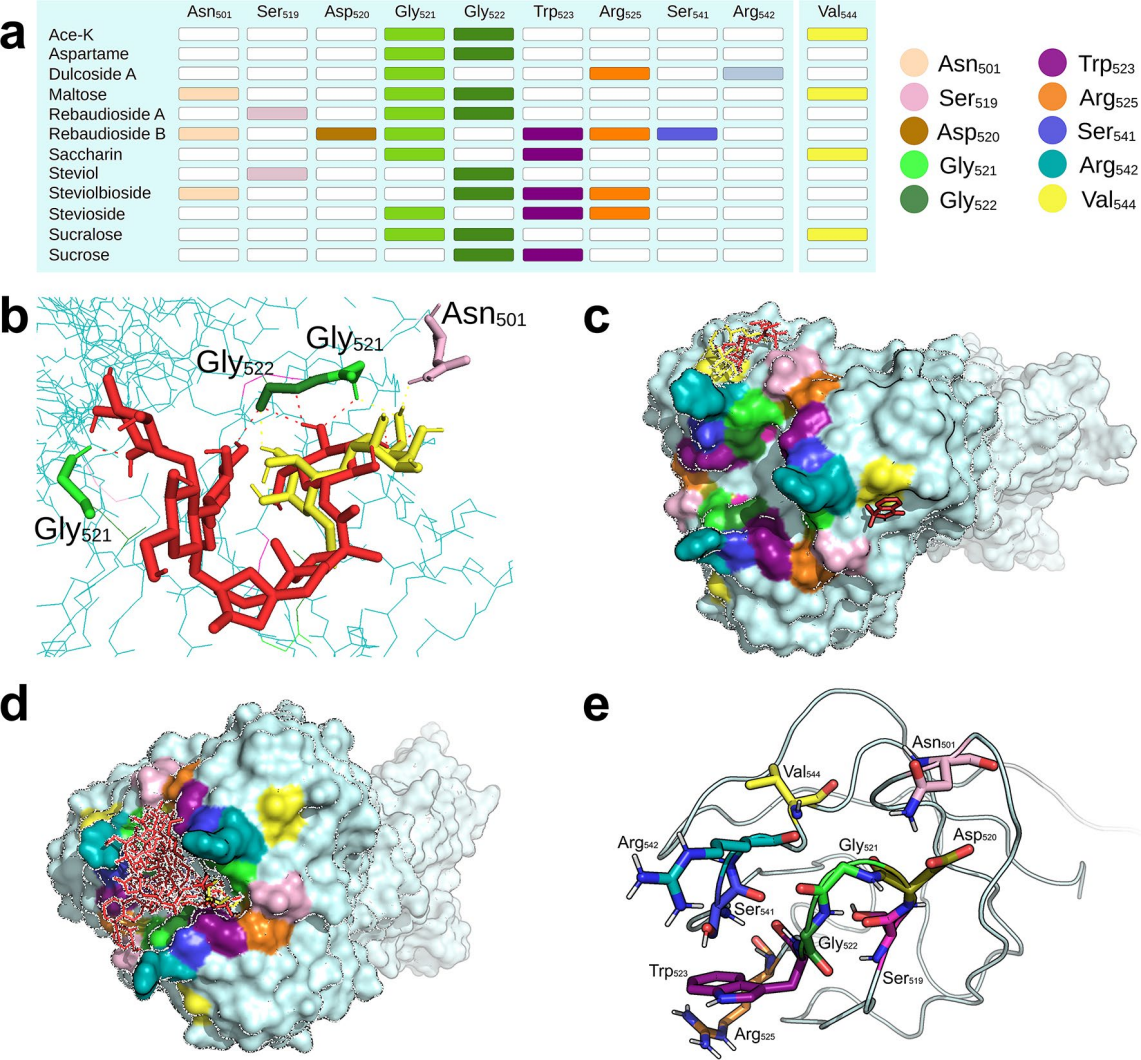
Docking analysis of gp17. (**a**) Summary table of the most stable poses between selected NNS and the fiber protein gp17 of phage T7. All NNS bound at least one core residue (Gly_521_ or Gly_522_). Ace-K, maltose, saccharin, and sucralose could bind the catalytic residue Val_544_, although not with the lowest binding energies; the separation of the box representing this residue remarks such a feature. (**b**) Localization of rebA (red) and maltose (yellow) on the pocket G521 of gp17. The natural substrate of gp17, maltose, binds Asn_501_, Gly_521,_ and Gly_522_. The NNS rebA also binds Gly_521_ and Gly_522_, directly overlapping with maltose’s pose and, thus, suggesting that the two molecules compete for the same pocket. (**c**) Surface visualization of gp17 with the position of the NNS (in red) and the natural sugars maltose (in yellow) and sucrose (in lilac). To note that neither the NNS nor maltose bound the catalytic residue Val_544_ in their most stable poses, and all the sweeteners clustered in the area of the protein. (d) Position of the NNS that bound to the catalytic residue Val_544_ at intermediate binding energies. In panels (**c**) and (**d**), the protein’s surface is color-coded according to the residues indicated on the bottom left of the figure.

The most stable conformation between the tested NNS and T7 gp17 clustered around residues Gly_521_ and Gly_522_, a pocket that was herein named locus G521 for convenience (Fig. 3a). In particular, rebA could overlap a binding pocket with maltose (Fig. 3b). The residues within locus G521 (Fig. 3c) formed a valley at the bottom of Arg_542_ in proximity but distinct from the catalytic residue Val_544_, which is located instead on the stem side of gp17 (Fig. 3d). In particular, only a subset of the NNS (ace-K, aspartame, and sucralose) bound to both residues; others (saccharin, steviol, and sucrose) established connections with either Gly_521_ or Gly_522_ and another residue, while the majority (dulcA, maltose, rebA, rebB, SB, and stevioside) bound more than two residues beside either Gly_521_ or Gly_522_. RebB and SB were the NNS that established the highest number of bonds with gp17: seven and five, respectively; consequently, these NNS had the most stable binding energies. Apart from ace-K and saccharin, all the other NNS had binding energies that were more stable than those of maltose. Although the most stable conformations between the tested NNS and gp17 were located within G521, ace-K, maltose, saccharin, and sucralose could directly bind the catalytic residue Val_544_^39^ (Fig. 1e), suggesting that these NNS have the potential to directly interfere with gp17’s recognition of *E. coli* strain K-12 and B moieties.

### Binding of rebA to phage KP32 gp31

**Fig. 4:**
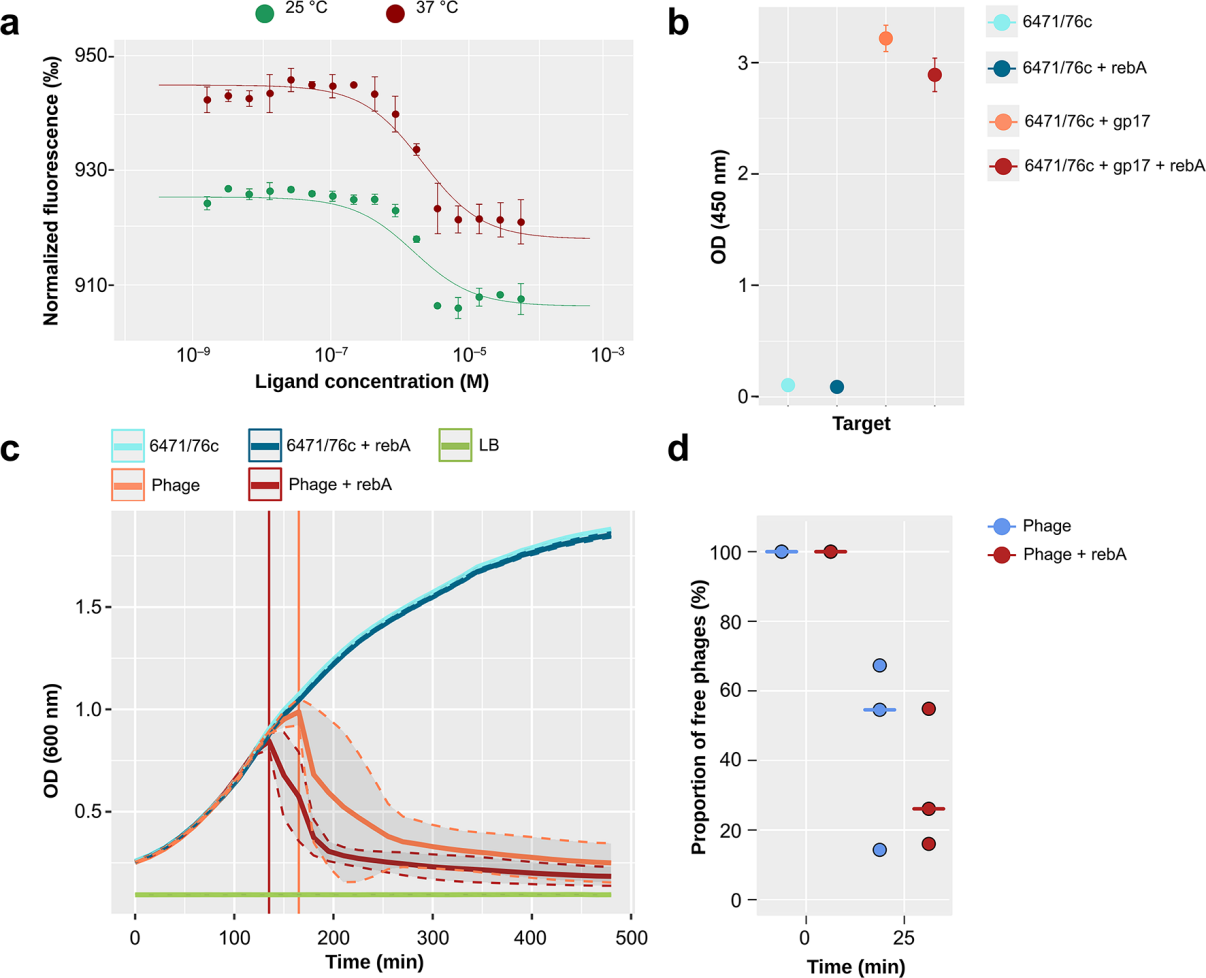
Binding analysis. (**a**) Association profile for the adsorption between KP32 gp31 and rebA at 25 (green) and 37 (red) °C; mean of three replicates with standard deviation. (**b**) Addsorption of gp17 to *Y. enterocolitica* serotype 6471/76c in the presence or absence of rebA. (**c**) Lysis rate *Y. enterocolitica* 6471/76c in the presence or absence of rebA. The plot reports the changes in OD_600_ measured with *Y. enterocolitica* 6471/76c and phage φYeO3-12. The phage was exposed (red line) or not (orange line) for 20 h to rebA. The growth of the bacteria alone in the presence (blue line) or absence (cyan line) of rebA is reported as mock infection control. The signal produced by LB alone (green line) is given as a reference. The beginning of the bacterial mass decline is depicted by vertical lines for the phage alone (orange, 165 min) and phage exposed to rebA (red, 135 min). The shaded areas represent one standard deviation from the mean of three replicates. (**d**) Quantification of the fraction of free phages in the presence or absence of rebA. The number of phages not adsorbed to their hosts are titrated at specific time points until the latent time is reached after 25 min. The phages were exposed (red) or not (blue) prior to the titration. The plot depicts the ratio of the PFU/mL at the beginning and end of the experiment. The median of the three replicates values is shown.

Microscale thermophoresis (MST) was performed using recombinant gp31 to test whether NNS could effectively bind to this protein, as suggested by the in silico analysis. Recombinant gp31 was consistent with its natural counterparts, showing a molecular mass of 21.6 kDa as previously reported^42^ (Supplementary Fig. 1a). MST analysis showed that the titration of gp31 (20 nM) against a gradient formed by a 1:2 serial dilution of rebA (50 µM to 1.5 nM) provided a K_d_ of 1.41 ± 0.48 µM at 25 °C and 1.88 ± 0.58 µM at 37 °C (Fig. 4a and Supplementary Fig. 2).

To confirm the binding of rebA to gp31 and assess whether such binding could affect the activity of this protein, we carried out an enzymatic assessment using a comparative test with the disaccharide chromogenic substrate *RedStarch*™ as a surrogate for maltose, gp31’s natural substrate. Our results indicated that, in the presence of rebA, gp31 showed a higher processivity than the control: gp31 alone had an activity rate of 3.07 ± 0.086 U/mL while gp31 exposed to rebA had an activity rate of 4.64 ± 0.449 U/mL, corresponding to an increment of 51.14% (Student’s t-test p-value = 0.004). We used the protein-solving buffer as a negative control and we observed no differences in the runs performed with or without rebA (data not shown), indicating that this NNS had not hydrolytic activity toward *RedStarch*™.

### Binding of rebaudioside A to *Yersinia* phage φYeO3-12 gp17

To indirectly assess the binding between rebA and gp17, we used an adsorption assay performed according to the protocol described by Filik et al.^42^. We used gp17 carrying a maltose binding protein conjugated to a histidine (His) tag (H/MTFP-gp17) whose purity, analyzed by SDS-PAGE, was greater than 95% (Supplementary Fig. 1b) with an estimated molecular mass of 113 kDa in accordance with previous measurements^42^. In the assay, the light signal at a wavelength of 450 nm (OD_450_) was proportional to the amount of gp17 adsorbed to immobilized bacterium due to the use of peroxidase-conjugated anti-His antibodies. The results showed that rebA exposure could decrease the adsorption of gp17 to its host compared to non-exposed controls: the OD_450_ of gp17 exposed to rebA was 2.887 ± 0.090 compared to 3.216 ± 0.184 of gp17 alone, corresponding to a slightly significant decrease of 10.2% (Student’s t-test p-value = 0.049) (Fig. 4b).

To further test the effect of rebA exposure on the phage activity, we assayed the phage-driven *Y. enterocolitica* 6471/76-c lysis by measuring the OD_600_ of bacterial cultures, assuming that the bacterial mass, assayed by optical density, would be inversely proportional to the lysis rate. Phages φYeO3-12 pre-incubated with rebA for 20 h displayed a faster bacteriolysis rate than the unexposed controls: the OD_600_ of rebA-exposed phages started to decrease at around 135 min post-infection (p.i.), whereas unexposed phages showed at 165 min p.i. (Fig. 4c). To exclude a direct effect of rebA on the growth of bacteria, we grew *Y. enterocolitica* 6471/76-c in the presence or absence of this NNS without φYeO3-12 phages. We observed no significant changes in the growth rate of the rebA-exposed bacteria over the unexposed controls, suggesting that the aforementioned OD_600_ decline was not due to the direct NNS influence on bacterial growth but by the indirect NNS-mediated alteration on phage infectivity.

We observed the same effect in the phage titration assay employing RTD. The phage titer in the sample with rebA was over three times higher than in the absence of this NNS: 13.00 ± 0.29 × 10^3^ plaque-forming units per milliliter (PFU/mL) against 4.14 ± 0.58 × 10^3^ PFU/mL (Mann-Whitney U test p-value = 0.036). The titration assay confirmed the increased phage predisposition to infect bacteria due to rebA. Additionally, we performed a preliminary measurement of the fraction of free phages upon exposure to rebA. After 25 min, the fraction of free phages was in median (IQR) 59.7 (40.8–60.5) percent when the viruses were not exposed to rebA compared to 53.0 (46.8–54.8) percent with pre-treated phages (Fig. 4d). Thus, the results suggested that exposure to rebA determined a 11.2% increase in phages bounded to their hosts compared to the untreated controls. Nonetheless, these results must be considered only preliminary since there was no statistical difference between the two rebA exposure groups (Mann-Whitney U test p-value = 0.700).

## Discussion

NNS are widely used in food products and other commercial items such as mouthwashes and vitamin supplements^25^. An increasing number of scientific reports have shown that the consumption of these sweeteners is associated with bacterial dysbiosis that, in turn, is linked to a series of health conditions ranging from metabolic syndrome to cancer^43^. In particular, stevia is gaining popularity because it is a natural sweetener and, therefore, well suited to the bio-organic food market^31^. Stevia comprises several steviol glycosides, with rebA being among the most abundant^29^.

The effect of NNS on phages has been neglected even though bacterial viruses are key elements in shaping bacterial genome diversity^4,44^. Thus, the present study’s purpose was to provide proof-of-principle for the concept that NNS can bind to phages and alter the viral infectivity process. We were interested in addressing the role of rebA in the infection of *Y. enterocolitica* and *K. pneumoniae *due to the medical and veterinary importance of these zoonotic bacteria^15^.

We conducted an in silico analysis of the binding between viral proteins involved in the infection process and the most common NNS, together with preliminary in vitro experiments to measure the variation in phage infectivity upon exposure to rebA. In particular, we focused our investigation on the possible rebA effect on the hydrolytic activity of baseplate proteins, the adsorption to bacterial surface moieties, and the overall infectivity of whole phages. Our docking analysis indicated that NNS could bind to viral proteins, specifically gp31 derived from *K. pneumoniae* phage KP32 and T7 gp17, suggesting that the sweeteners investigated in this study might have the potential to interfere with the protein’s activity.

We confirmed this in silico exploration employing MST, showing that the affinity between gp31 and rebA increased by 25% from environmental (25 °C) to physiological temperatures (37 °C). We interpreted these data by assuming that rebA could bind gp31 in a temperature-dependent manner. In addition, we assessed the possible impact of rebA on gp31 by measuring the hydrolytic activity of this protein in the presence of this NNS. Unexpectedly, we observed a significant increase of approximately 51% in enzyme activity in rebA-exposed gp31 compared to unexposed controls. We concluded that rebA could stabilize gp31, which is also a structural viral protein, somehow enhancing its enzymatic activity. Such a result is compatible with previous studies reporting on the stabilization effect of sucrose on phage capsids^45–47^. In light of our docking analysis, it might be speculated that rebA could bind to pockets not overlapping with maltose, such as K57 or S144, exerting a structural stabilization on gp31. These results also highlighted the possibility of artificially boosting gp31’s activity to increase biofilm dissolution, a feature that can have applications in the medical field and food processing.

The effect of rebA on gp17 was more complex to interpret. Our docking analysis was based on the fiber protein gp17 of phage T7 because high-resolution structural data for φYeO3-12 gp17 are missing but it has been shown that these two proteins are highly related^48^. In particular, our previous studies on the TTPA group revealed that gp31 from KP32 is a homolog to gp11 of φYeO3-12 since these proteins share 59% amino acid homology and both possess hydrolytic activity toward maltose^49^. The docking analysis we performed herein indicated that Asp_520_ and Val_544_ were important in recognizing *E. coli* B and K-12, respectively, with the additional involvement of Ala_518_^40^. Moreover, it has been reported that the anti-parallel β-sheet between residues 57 and 99 should be involved in substrate recognition, with Asp_122_-X-Asp_124_ and Asp_151_-X-Glu_153_being part of the catalytic site^9^.

We indirectly confirmed the binding of rebA to recombinant gp17 by measuring the adsorption of this protein to the host *Y. enterocolitica* 6471/76-c, observing a slight decrease of 10% in the adsorption rate of rebA-exposed proteins compared to unexposed controls. Conversely, we quantified the phage-driven bacteriolysis employing phages obtained from bacterial lysates, observing that exposure of phage φYeO3-12 to rebA accelerated *Y. enterocolitica* lysis by about 30 min compared to unexposed phages.

The inhibition of adsorption to the host of rebA-exposed recombinant proteins was expected in light of our in silico data, which suggested the possible binding of rebA to the residues involved in recognizing O-specific polysaccharides within gp17^42^. Conversely, the enhancement of bacteriolysis upon exposure to rebA was unexpected. Nonetheless, it must be considered that the conditions of these assays were significantly different. The adsorption test was based on recombinant proteins and bacteria were immobilized on a surface, whereas in the lysis test, bacteria were in a planktonic state. It has been suggested that planktonic bacteria can be infected more easily than immobilized ones^50^, a feature that might explain the different results. Moreover, the adsorption test was based on a single recombinant protein, while the lysis assay used whole virions, suggesting that other proteins or factors might be involved in the infection process apart from gp17 and rebA could facilitate such a phase. Our observation, albeit only preliminary, that exposure to rebA might decrease the number of free phages compared to unexposed controls might support the case for a rebA-induced increased bacteriolysis rate.

Thus, the seemingly contradicting results, while requiring further investigation with highly purified phage particles to determine the underlying mechanism of action, can be explained by hypothesizing that, on the one hand, rebA might compete with gp17 in the recognition of *Y. enterocolitica*surface moieties, and, on the other hand, rebA might stabilize the overall virion structure, superseding the expected inhibition. Such conjecture is based on the stabilizing effect shown by sucrose^36^ but it is further supported by recent evidence demonstrating that rebA, alongside other steviol glycosides, had an emulsifying effect upon soy proteins mediated by van der Waals and hydrogen bonds^51^. Assuming the presence of virion aggregates in suspensions^52^, it might be assumed that rebA might resolve these assemblages effectively increasing the viral titer, as reported herein.

The present work had some limitations. It was conceived to provide a proof-of-concept to assess the possible role of NNS on phage infectivity; hence, additional investigations are required to better understand the mechanism of action of NNS on phage biology and the repercussions on bacterial communities. The lack of high-resolution tridimensional structure for phage φYeO3-12 gp17 limited the observation of the details of the binding of NNSs to this protein; thus, the determination of the fine structure of this protein would provide valuable information upon the understanding of the influence of NNSs on phage biology. Moreover, the employment of virions obtained from lysates rather than purified ones might have affected the results involving the use of whole viruses, a feature that was particularly relevant in the quantification of the fraction of free phages. It is possible that debris from the lysis co-present with phage φYeO3-12 might have interfered with the infection process. Therefore, a description of the influence of phage infection in the presence of rebA or other NNS must be carried out employing purified phages to reduce the bias of the results.

Nonetheless, our exploratory investigation established that the NNS rebA has the potential to bind phage proteins involved in the infection process and, indeed, alter the activity of these proteins compared to unexposed controls.

## Conclusions

The present study sought to investigate the possible role of NNS on the biology of phages, a feature that has been overlooked in the scientific literature. We focused our analysis on the effect of stevia’s main compound rebA on the activities of *Y. enterocolitica* phage φYeO3-12 gp17 and *K. pneumoniae* phage KP32 gp31. Our results showed that, based on in silico analysis, both gp17 and gp31 had the potential to bind rebA. Additional in vitro analysis confirmed the binding of rebA to gp31 and an increase in the enzymatic activity of this protein compared to unexposed controls. Moreover, rebA decreased the adsorption of recombinant gp17 to immobilized hosts but increased the lysis rate of whole φYeO3-12 phages. These results supported the concept that NNS might alter the infectivity of phages.

## Methods

### Docking

The tridimensional structures of the target proteins were retrieved from the *Protein Data Bank* (PDB)^53^, whereas those of the ligands were retrieved from *Chemspider* (http://www.chemspider.com/). Areas and volumes of the ligands were computed with *ChimeraX*^54^ by generating a surface model and applying the *Measure Volume and Area* tool. The ligands’ isoelectric points (pI) were obtained through the *Expasy* website, *Compute pI/MW* tool (https://web.expasy.org/compute_pi/). The molecular weights (MW) of the ligands were obtained from *Chemspider*.

Targets and ligands were prepared for docking using *AutoDockTools*ver. 1.5^55^ by removing water molecules and adding both hydrogens and Kollman’s charges to the structures. Docking was performed using *AutoDock Vina* ver. 1.5^56^, which provided nine conformations (poses) for each ligand. Graphical rendering of the poses was obtained with *PyMol*ver. 2.5^57^. Although the tridimensional (3D) structure of φYeO3-12 gp17 (fiber protein) and gp11 (tail tubular protein) are still missing, they share considerable identity with *E. coli* phage T7 gp17 and *K. pneumoniae* phage KP32 gp31, respectively, which have been characterized by X-ray cristallography^41^.

### Phage proteins purification

Recombinant gp31 and gp17 labeled with maltose binding protein (MBP) moieties were produced in a bacterial expression system as previously described^9,42^. Both proteins contained His-tag and were purified using two rounds of nickel-affinity chromatography. Proteins were eluted with 20 mM Tris/HCl buffer, pH 8.0, 300 mM NaCl, 5% glycerol, and 5 mM β-mercaptoethanol containing 250 mM of imidazole. Before the second round, the eluted protein fraction was precipitated with 0.65 g/ml ammonium sulfate, harvested by centrifugation (20,000 g, 4 °C, 45 min), and resuspended. To remove the MBP and the His-tag, the protein solution was digested by TEV protease overnight at 4 °C. After digestion, the tag-free protein appeared in the unbound fraction of proteins (flow-through) during nickel-affinity chromatography. The flow-through fraction was again precipitated by adding 0.65 g/ml ammonium sulfate overnight at 4 °C and harvested by centrifugation (20,000 g, 4 °C, 45 min). The Knauer system of chromatography was used for protein purification by affinity chromatography. The system was controlled using the Purity Chrome software. The stage of enzymatic cleavage was omitted to obtain gp17 containing MBP and His-tag needed for the ELISA test. The proteins were analyzed by SDS-PAGE electrophoresis on 12% polyacrylamide gels (*Bio-Rad*) and molecular weights were evaluated against the *BlueStar Plus Prestained Protein Marker* (*Nippon Genetics*) mass ladder. The proteins’ purity was estimated by densitometric analysis with the *GeneSnap* software ver. 7.09 (https://www.snapgene.com/*).*

### Microscale thermophoresis (MST)

Purified recombinant gp31 was labeled using *Protein Labeling Kit RED* 2nd generation according to the manufacturer protocol (*NanoTemper Technologies*). The labeled proteins were quantified by *Nanodrop 1000* (*ThermoFisher*) and then titrated against a concentration gradient of rebA (*Merck*) in PBS supplemented with 0.005% v/v of *Tween20*^®^ (*Merck*) at a temperature of 25 and 37 °C. The molecular interactions between the protein and the ligands were measured within premium MST glass capillaries (*NanoTemper Technologies*) in a *Monolith NT 115* instrument (*NanoTemper Technologies*) at an LED power of 40%. *MO.Affinity Analysis v2.2.4* software (*NanoTemper Technologies*, https://shop.nanotempertech.com/en/moaffinity-analysis-software-unlimited-licenses-34*)* was used to process the data and provide a coefficient of dissociation (K_d_) to measure the association affinity between protein and ligand.

### Hydrolytic activity assay

The impact of rebA on the hydrolytic activity of *Klebsiella* phage KP32 gp31 was assayed using a chromogenic substrate as previously described^48^ with some modifications. Briefly, 200 µL of 2% w/v *RedStarch*™ (*Megazyme*) in 0.5 M KCl was mixed with 50 µL of gp31 (4.673 µM) in the presence or absence of 50 µL (14.019 µM) of rebA. In the sample without addition of the rebA 50 µL of protein solvent was added to maintain same volumes for reaction, 100 µL of protein solvent without gp31 and 50 µL with addition of 50 µL (14.019 µM) of reb A served as control. Samples were incubated at 20 °C for 72 h. The reaction was stopped by adding 500 µL of 96% ethanol, followed by incubation for 10 min at room temperature and centrifugation (1000 ×g, 10 min). Afterward, 250 µL of supernatant was collected, and absorbance was measured at a wavelength of 510 nm. The enzyme activity was calculated based on a protocol for assay of α-amylase using *RedStarch*™ according to the manufacturer’s recommendations.

### Adsorption test and lytic performance

The impact of the NNS rebA on the adhesive properties of phage φYeO3-12 gp17 was assayed by enzyme-linked immunosorbent assay (ELISA) as previously described^42^. Colony-forming units per milliliter (CFU/mL) and plaque-forming units per milliliter (PFU/mL) were evaluated using the protocol described by Kropinski et al.^58^. To assay the impact of rebA on φYeO3-12 phage infectivity performance in real-time, the bacterial mass was measured with a microplate reader Tecan® SPARK (*Tecan*) as follows. An overnight culture of *Y. enterocolitica* 6471/76-c (courtesy of Professor Skurnikin from the Department of Bacteriology and Immunology, Helsinki One Health, Finland) in Luria-Bertani (LB) medium (10 g/L tryptone, 10 g/L NaCl, 5 g/L yeast extract) was diluted in LB medium to 2.6 × 10^8^ CFU/mL. Then, 200 µL of the suspensions were dispensed on the wells of the 96-well plate (*NEST 96 – TC Treated*). The bacteria were exposed to 10 μL of phage φYeO3-12 at a density of 40 PFU/mL pre-incubated or not with rebA for 20 h at 4 °C (the concentration of rebA was 0.05 mM). Bacteria exposed or not to the same amount of rebA in the absence of phages were used as mock infection controls. The samples were incubated at 28 °C for 10 h (210 rpm) and the bacterial density was monitored by measuring the optical density of the samples at a wavelength of 600 nm (OD_600_) every 15 min. Routine test dilution (RTD) was carried out using 50 μL of an overnight culture of *Y. enterocolitica* 6471/76-c supplemented with fresh LB medium to achieve logarithmic growth. The suspension was diluted to approximately OD_600_ 0.15. Afterward, 1 mL of a 1:10 serial dilution of the suspensions ranging from 10^0^ to 10^–3^ was spread on LB agar plates. Each plate was then supplemented with phages with or without rebA. LB medium with or without rebA were used as controls. The plates were incubated at 28 °C for 24 h. After incubation, the phage load, expressed in PFU/mL was calculated.

The phage adsorption test was performed following the protocol described by Kropinski et al.^58^. with slight modifications. Briefly, *Y. enterocolitica* 6471/76c were grown to mid-logarithmic phase in LB medium and diluted to an OD_600_ of 0.1 and then inoculated with phage φYeO3-12 at density of 10^4^PFU/mL pre-incubated or not with rebA (0.05 mM) for 20 h. The suspensions were incubated at 28 °C and probed by withdrawing an aliquot of 50 µL every five minutes up to 25 min, which corresponded to the latency of this phage^59^, to determine the number of phages not adsorbed to the host. The phage density for each probe was determined by standard double agar layer method^60^. The fraction of free phages was calculated as a ratio of the phage titer at the different time points over the number of phages without bacteria.

### Statistical analysis

All experiments were repeated three times, and mean values with standard deviation or median with interquartile range (IQR) were used as measures of data dispersion. Statistical analysis was performed using the *XrealStats* (https://real-statistics.com/) add-in for *Excel* software ver. 2021 (*Microsoft Inc.*). A two-sided Student’s t-test performed analysis of variance and Mann-Whitney U test was used to assess differences between groups. Statistical significance was set for p-values below 0.05). Visualization of the results was obtained with *R* ver. 4.3.3^61^.

## Electronic supplementary material

Below is the link to the electronic supplementary material.

Supplementary Material 1

Supplementary Material 2

Supplementary Material 3

## Data Availability

Supplementary information is provided within the manuscript.
