# Supplementary material for: The non-nutritive sweetener rebaudioside a enhances phage infectivity
Source: Sci Rep. 2025 Jan 8;15:1337. doi: 10.1038/s41598-025-85186-w (PMC11711195; doi:10.1038/s41598-025-85186-w)
Supplement: Supplementary file 3 — Supplementary Material 3 [file 41598_2025_85186_MOESM3_ESM.docx]

**Supplementary Figure 1. Production of recombinant phage proteins.**

a) SDS-PAGE of gp31 after nickel-affinity chromatography (lane 1), and SEC purification (lane 2: 0.04 nM; lane 3: 0.22 nM). b) SDS-PAGE of gp17-MBP-His after nickel-affinity chromatography (lane 1), and SEC (lane 6: 0.05 nM). Lanes 3 to 5 are subsequent fractions collected during purification on SEC. The molecular weight ladder is provided in lanes 0.

**Supplementary Figure 2. Microscale thermophoresis of gp31.**

a) MST traces between gp31 and rebA at 25 °C. b) MST traces between gp31 and rebA at 37 °C. In panels (a) and (b), the plot reports the normalized fluorescence from 16 capillaries (positions) containing the same amount of protein but increasingly more diluted amounts of rebA from 50 μM (position 1, darker color) to 1.5 nM (position 16, lighter color). The shaded areas represent one standard deviation from the mean of three replicates.
